# Analysis of Maxi-K alpha subunit splice variants in human myometrium

**DOI:** 10.1186/1477-7827-2-67

**Published:** 2004-09-21

**Authors:** Michael Curley, John J Morrison, Terry J Smith

**Affiliations:** 1 National Centre for Biomedical Engineering Science, National University of Ireland, Galway, Galway, Ireland; 2 Department of Obstetrics and Gynaecology, National University of Ireland, Galway, Clinical Science Institute, University College Hospital Galway, Newcastle Road, Galway, Ireland

## Abstract

**Background:**

Large-conductance, calcium-activated potassium (Maxi-K) channels are implicated in the modulation of human uterine contractions and myometrial Ca^2^^+^ homeostasis. However, the regulatory mechanism(s) governing the expression of Maxi-K channels with decreased calcium sensitivity at parturition are unclear. The objectives of this study were to investigate mRNA expression of the Maxi-K alpha subunit, and that of its splice variants, in human non-pregnant and pregnant myometrium, prior to and after labour onset, to determine whether altered expression of these splice variants is associated with decreased calcium sensitivity observed at labour onset.

**Methods:**

Myometrial biopsies were obtained at hysterectomy (non-pregnant, NP), and at Caesarean section, at elective (pregnant not-in-labour, PNL) and intrapartum (pregnant in-labour, PL) procedures. RNA was extracted from all biopsies and quantitative real-time RT-PCR was used to investigate for possible differential expression of the Maxi-K alpha subunit, and that of its splice variants, between these functionally-distinct myometrial tissue sets.

**Results:**

RT-PCR analysis identified the presence of a 132 bp and an 87 bp spliced exon of the Maxi-K alpha subunit in all three myometrial tissue sets. Quantitative real-time PCR indicated a decrease in the expression of the Maxi-K alpha subunit with labour onset. While there was no change in the proportion of Maxi-K alpha subunits expressing the 87 bp spliced exon, the proportion of alpha subunits expressing the 132 bp spliced exon was significantly increased with labour onset, compared to both non-pregnant and pregnant not-in-labour tissues. An increased proportion of 132 bp exon-containing alpha subunit variants with labour onset is of interest, as channels expressing this spliced exon have decreased calcium and voltage sensitivities.

**Conclusions:**

Our findings suggest that decreased Maxi-K alpha subunit mRNA expression in human myometrium at labour onset, coupled to an increased proportion of Maxi-K channels expressing the 132 bp spliced exon, may be linked to decreased Maxi-K channel calcium and voltage sensitivity, thereby promoting enhanced uterine activity at the time of labour.

## Background

The regulatory mechanisms for uterine smooth muscle contractility during human pregnancy and labour are poorly understood. Such information is essential to understanding the clinical problems associated with human parturition and particularly preterm or premature labour. It is clear however that the myometrium is transformed from a state of relative quiescence during pregnancy, to one of maximal contractile activity at the time of labour. It is also established that the state of contractility of uterine smooth muscle is intrinsically linked to cell membrane ion channel activity [1,2].

Potassium (K^+^) channels are functionally important in the regulation of smooth muscle tone [3]. Among the diverse family of K^+ ^channels, large-conductance, calcium-activated K^+ ^(Maxi-K, also known as BK_Ca_) channels are the predominant K^+ ^channels in myometrium, and thus have been implicated in the control of cellular excitability [4]. While evidence for an important role of Maxi-K channels is not particularly strong, it is thought that they play a pivotal role in the modulation of uterine contractility and myometrial calcium homeostasis. Pharmacological inhibition of Maxi-K channels, by the specific channel blocker iberiotoxin, increases contractile activity in human uterine tissue [5], whereas compounds that promote Maxi-K channel opening, such as NS1619, have a potent relaxant effect on pregnant human myometrium [6]. Structurally, Maxi-K channels are tetramers of a pore-forming α subunit of the *slo *gene family, and a regulatory β subunit [7-10]. The α subunit comprises 7 transmembrane regions (S0-S6) and 4 intracellular hydrophobic domains (S7-S10) [11]. The β subunit is a structurally unique, membrane-spanning protein that contributes to channel gating and pharmacology [12]. The α subunit is encoded by a single gene. However, it achieves molecular diversity by extensive alternative splicing of its gene transcript at several sites [7,13-15], which generates Maxi-K channel variants. There is a substantial body of evidence indicating that alternate splicing of the maxi-K transcript plays a major role in regulating potassium channel conductance [7,15]. These data include evidence for splice variation effecting calcium and voltage sensitivity, surface expression, and sensitivity to protein phosphorylation of the maxi-K channel [16,17]; [18]. Alternative splicing of the maxi-K channel α subunit is considered to be a molecular mechanism by which the channel is able to adjust and tune its response to a variety of regulatory and conductance requirements. Further evidence of the role of alternate splicing of the maxi-K transcript in altering maxi-K protein function in myometrium is provided by the finding of up-regulation of maxi-K splice variants known to alter channel current through alterations in calcium and voltage sensitivity in pregnant mouse myometrium [19]. What initiates alternative splicing of the α subunit transcript is incompletely understood, however there is evidence that expression of different alternatively spliced transcripts can be hormonally induced [20,21].

It appears that expression of different pore-forming α subunit isoforms, with associated regulatory β subunits, occurs in a tissue-specific manner, thereby providing functional specificity [22]. Maxi-K channels have been identified both in human non-pregnant [23] and pregnant [24] myometrium. For animal myometrial tissues, the data outlining Maxi-K α subunit mRNA expression in relation to labour are conflicting [19,25,26]. For human myometrium, it has more recently been reported that protein expression of both α and β subunits is down-regulated with labour onset [27].

Although multiple alternatively spliced exons of the Maxi-K α subunit have been identified [10,19,21,28], there is no information available to date pertaining to expression of α subunit splice variant mRNA transcripts in human myometrium during pregnancy or at the time of labour. Because phosphorylation sites can be introduced into the channel protein via alternatively spliced exons [19], alternative splicing may represent an important control mechanism regulating Maxi-K channel function during pregnancy and at labour. The aim of this study was to investigate the expression of alternatively spliced exons of the Maxi-K α subunit transcript in non-pregnant myometrium and in pregnant myometrium, prior to and after labour onset using quantitative real-time PCR.

## Methods

### Patient recruitment and tissue collection

Patient recruitment took place in the Department of Obstetrics and Gynaecology, University College Hospital Galway (UCHG), Ireland, between October 2001 and August 2002. The study was approved by the Research Ethics Committee, UCHG, and recruitment was carried out by provision of information sheets and obtaining written informed consent.

Biopsies of myometrium were excised from the midline of the upper lip of the uterine incision made at caesarean section, at elective (pregnant not-in-labour, PNL; n = 8) and intrapartum (pregnant in-labour, PL; n = 7) procedures. The mean age of the women was 33.3 years (range 26–42) of whom four were primagravida and eleven were multigravida. All women were delivered between 37 and 41 weeks gestation. There was no significant difference between those undergoing elective or emergency caesarean section in terms of age, parity or gestation. Women who had received prostaglandins or oxytocin were excluded from the study. Reasons for emergency section included breech presentation, previous caesarean section and abnormal foetal position. The criteria for inclusion in the intrapartum group were regular spontaneous uterine contractions, effacement of the cervix, and cervical dilatation >3 cm prior to caesarean section.

Samples of non-pregnant myometrium (NP; n = 7) were excised from the body of the uterus of hysterectomy specimens from pre-menopausal women. The mean age of women undergoing hysterectomy was 42.5 years (range 34–48). Women with malignant conditions, and those receiving exogenous hormone therapy (e.g. progestagens), were excluded from the study. Immediately upon removal, tissue samples were rinsed in sterile saline, snap frozen in liquid nitrogen and stored at -80°C until RNA extraction.

### RNA preparation/Reverse Transcriptase-Polymerase Chain Reaction

RNA was isolated from frozen tissue by homogenisation in TRIzol^® ^Reagent (Life Technologies, Paisley, UK) [29]. RNA concentration was determined by absorbance at A_260_. To eliminate any residual contaminating genomic DNA, all RNA samples were DNase-treated with the DNA-free™ DNA removal kit (Ambion, Huntingdon, Cambridgeshire, UK), as previously described [30]. RNA concentration was measured again by absorbance at A_260_, after removal of DNA, and adjusted to a final concentration of 500 ng/μL.

Reverse Transcriptase-Polymerase Chain Reaction (RT-PCR) was performed to check for mRNA expression of all potential spliced exons of the Maxi-K α subunit in non-pregnant and pregnant myometrium, prior to and after labour onset. Purified RNA samples were reverse transcribed using oligo (dT)_15 _primer and 200 IU M-MLV reverse transcriptase (Promega, Madison, WI, USA), as previously described [30]. PCR amplification was performed with 20 pmol of each specific oligonucleotide primer pair (Table 1), and 1.25 IU Taq DNA Polymerase (Promega, Madison, WI, USA) as previously described [30]. Primer pairs were designed to flank predicted splice sites, allowing spliced exon expression in these different regions to be assessed. PCR products were separated by electrophoresis on a 1.5% agarose gel and visualised after ethidium bromide staining by UV illumination. Bands identified were purified by gel extraction using Qiagen Gel Extraction kit (Qiagen, West Sussex, UK), and sent for sequencing (MWG-Biotech Ltd., Milton-Keynes, UK).

**Table 1:** Primer Pairs

Primer name	Primer sequence (5' – 3')	Primer Tm (°C)
Maxi 0F	CGGAGGCAGCAGTCTTAG	58.2
Maxi 0R	AAGAAAGTCACCATGGAGGAG	57.9
Maxi 1F	CTCCTCCATGGTGACTTTCTT	57.9
Maxi 1R	TTACAAGTGCACCGATGCTG	57.3
Maxi 2F	GGAAACCGCAAGAAATAC	53.1
Maxi 2R	ACCTCATGGAGAAGAGGTTG	57.3
Maxi 3F	GGTCTGTCCTTCCCTACTGT	59.4
Maxi 3R	CAAAGATGCAGACCACGACA	57.3
Maxi 4F	GTGCCAGCAACTTTCATTAC	55.3
Maxi 4R	TCAGGGTCATCATCATCGTC	57.3
Maxi 5F	ACAGCATTTGCCGTCAGTG	56.7
Maxi 5R	GGTCCGTCTGCTTATTTGCT	57.3
β-actin F	CAACTCCATCATGAAGTGTGAC	55.8
β-actin R	GCCATGCCAATCTCATCTTG	59.3

### Splice variant-specific cDNA synthesis

Splice variant-specific cDNAs were prepared for each RNA sample using reverse primers as shown in Table 2. 3 μg of purified RNA (1 μg for β-actin) was reverse transcribed to cDNA for each amplicon of interest using 500 nmol/L specific reverse primer and 200 IU M-MLV reverse transcriptase (Promega, Madison, WI, USA), as previously described [30]. These cDNAs were stored at -20°C until required for real-time PCR. New forward primers were designed upstream of the spliced exon sequences, to ensure that product size was approximately 200 bp, the optimum product length for use with hydrolysis TaqMan probes.

**Table 2:** Amplicon-specific primer and probe sequences

Primer name	Primer/Probe sequence (5' – 3')	Primer/Probe Tm (°C)
Conserved F	TGCACAAAGAGGTATGTCATCAC	58.9
Conserved R	GTTTGCTGTGGATGGGATGGA	59.8
Conserved Probe	6F * -CCCACTCGTCGCAGTCCTCCAGCAAGAAGA XT♣	68.9
132 bp splice F	ACGCTCAAGTACCTGTGGACCGT	64.2
132 bp splice R	TGTGGTTCCAGTTGAGTCACCA	60.3
132 bp Probe	6F-CTCCAGGGTGGAGTGATTGGCTGTATGTT XTCAC	71.5
87 bp splice F	CATCGCAAGTGATGCCAAAGAA	58.4
87 bp splice R	TCAACTGGCTCGGTCACAAGC	61.8
87 bp Probe	6F-TTGCAGCTAGATCACGCTATTCCAAAGATCCA XT	68.9
β-actin F	CAACTCCATCATGAAGTGTGAC	55.8
β-actin nested R	GTCAAGAAAGGGTGTAACGCA	55.4
β-actin Probe	6F-TGGCACCCAGCACAATGAAGATCAAATCA XT	70.3

* 6F = FAM reporter dye; ♣ XT = TAMRA quencher dye

### Synthesis of cDNA standards

Standards (1 × 10^9 ^to 1 × 10^4 ^cDNA copies, in 10-fold increments) were created for each spliced exon and conserved region amplicons, and for β-actin, to enable accurate quantitation of product-specific cDNA copy numbers. Amplicon-specific PCR products were generated by RT-PCR (as described above) using primers shown in Table 2. Products were purified using Qiagen PCR purification kit (Qiagen, West Sussex, UK), quantified by absorbance at A_260_, and ligated into TA cloning vector pCR^® ^2.1 (TA cloning^® ^kit, Invitrogen Ltd, Paisley, UK), according to manufacturers' instructions. Vector-ligated PCR products were transformed into One Shot^® ^TOP10 cells, which were plated on Luria-Bertani (LB) agar plates containing 50 μg/mL kanamycin antibiotic, and incubated overnight at 37°C. Plasmid templates containing inserts in the desired orientation to transcribe sense RNA, as determined by colony PCR, were linearized by HindIII digestion. 2 μL of digestion products were electrophoresed on 1% agarose gels and visualised to ensure complete plasmid linearisation.

Sense cRNA transcripts were generated by *in vitro *transcription using the MAXIscript™ *In vitro *Transcription Kit (Ambion, Huntingdon, Cambridgeshire, UK). 1 μg of linearized plasmid DNA was *in vitro *transcribed in a final volume of 20 μL containing 0.5 mmol/L each of ATP, CTP, GTP, and UTP, 2 μL 10X Transcription buffer, and 2 μL T7 Enzyme mix for 1 hr at 37°C. After transcription, samples were treated with DNA-free™ DNA removal kit (Ambion, Huntingdon, Cambridgeshire, UK) to remove plasmid DNA. The supernatant, containing purified cRNA, was pipetted onto pre-hydrated NucAway™ spin columns (Ambion, Huntingdon, Cambridgeshire, UK) to remove free nucleotides from the transcription reaction and further purify the cRNA samples. These columns were centrifuged at 1200 g for 2 min. Eluted cRNA concentration was determined by absorbance at A_260_. Copy number/μL of cRNA was calculated according to the following formula, available from the Roche Lightcycler™ website:



Once the total amount of cRNA copies/μL had been calculated, serial dilutions of cRNA standards were produced (from 1 × 10^9 ^cRNA copies/μL to 1 × 10^4 ^cRNA copies/μL, in 10-fold increments) for each product-specific cRNA molecule generated. Serially-diluted cRNA standards were reverse transcribed in a 20 μL final volume as described above, using transcript-specific reverse primers (Table 2), thereby generating product-specific cDNA standards. These cDNA standards were stored at -20°C until required for real-time PCR.

### Quantitative expression analysis using real-time PCR

Real-time PCR amplification was performed on the Lightcycler™ instrument using the Lightcycler™ FastStart DNA Master Hybridization Probes kit (Roche Diagnostics, Mannheim, Germany). Hydrolysis TaqMan probes were synthesized for each amplicon to be quantified (TIB MolBiol Syntheselabour, Berlin, Germany), and are shown in Table 2. Probes were designed with consideration taken for the design parameters outlined by Bustin [31]. Probes were resuspended in PCR-grade water to a working stock concentration of 4 μmol/L, and stored in the dark at 4°C. Prior to quantitative analysis, several titration experiments for cDNA, probe, primer, and MgCl_2 _concentration were performed to determine optimum reaction conditions for amplification. The following master mix of the reaction components was prepared to the indicated end-concentration: 10.6 μL water, 2.4 μL MgCl_2 _(3 mmol/L), 1.0 μL forward primer (0.5 μmol/L), 1.0 μL reverse primer (0.5 μmol/L), 1.0 μL specific probe (200 nmol/L) (see Table 2) and 2 μL Hybridization Master Mix. The master mix (18 μL) was aliquoted into Lightcycler™ glass capillaries (Roche Diagnostics) and 2 μL cDNA (samples and standards) was added to respective capillaries. Capillaries were centrifuged at 3000 rpm for 5 s, and loaded into the Lightcycler™ instrument.

The experimental protocol used for TaqMan probe quantitative analysis consisted of two stages: initial denaturation (95°C for 10 mins), followed by 45 cycles of denaturation (95°C for 0 s) and annealing/extension (59–61°C for 50 s). The annealing temperature used in each experiment was dependent on the melting temperature of both the primers and probe involved. Fluorescence data were acquired at the end of each annealing/extension cycle.

Data analysis was performed using Lightcycler™ Second Derivatives Method software. This method automatically determines the threshold cycle (C_T_) values for each individual sample using a software algorithm, which allows initial mRNA concentration in each sample to be accurately quantified based on the standards used. Using this method removes user influence, as well as any influence of background fluorescence on the data. The fluorescence display mode used was F1/F2, which is the optimal setting for use with hydrolysis TaqMan probes. PCR products were isolated from capillaries after each program had finished and were visualised by electrophoresis on 1.5% agarose gels.

### Statistical analysis

The SPSS computer software package was used for all statistical analyses (Statistical Package for the Social Sciences, v.10, SPSS Inc., Chicago, IL, USA). Multiple group comparisons were made using analysis of variance (ANOVA), which were followed by individual group comparisons using the Tukey HSD test, where appropriate.

## Results

### Analysis of expression of alternatively spliced exons of the Maxi-K α subunit in human myometrium

**Figure 1:**
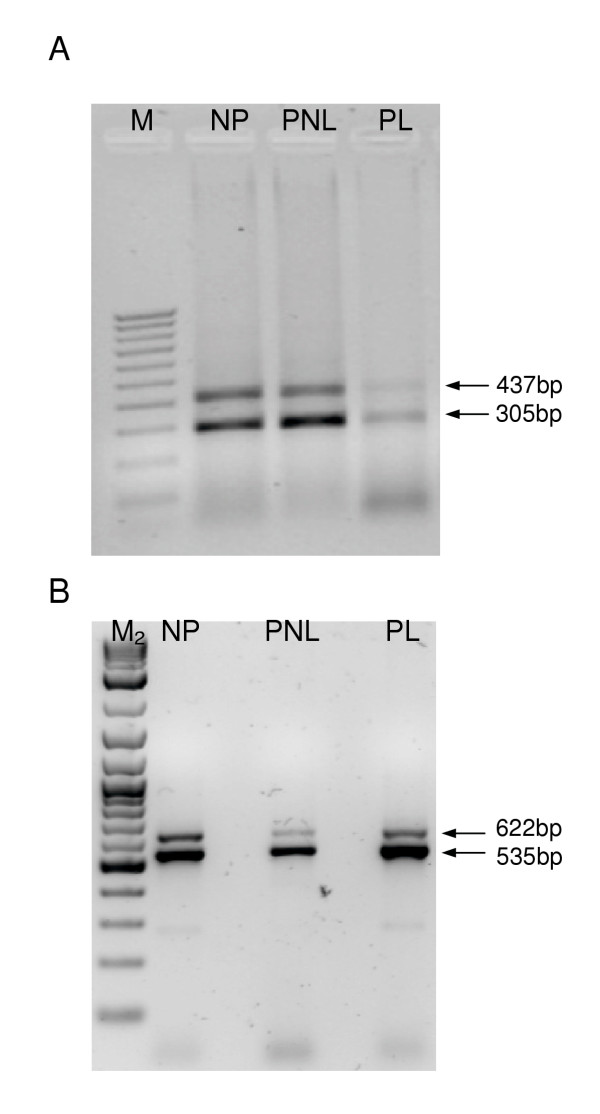
**Detection of alternatively spliced exon-containing RT-PCR products**. Ethidium bromide stained agarose gels (2%) showing (**A**) 132 bp spliced exon-containing (437 bp) and exon-less (305 bp) PCR products and (**B**) 87 bp spliced exon-containing (622 bp) and exon-less (535 bp) PCR products, in non-pregnant (NP), pregnant not-in-labour (PNL), and pregnant in-labour (PL) myometrial tissues. M = 100 bp marker (Promega, US); M_2 _= 2-log ladder (New England Biolabs Inc., UK).

RT-PCR analysis of the Maxi-K α subunit gene, using primers designed to flank predicted splice sites, produced a variety of bands in samples of non-pregnant (NP), pregnant not-in-labour (PNL) and pregnant in-labour (PL) myometrium (data not shown). From this analysis, only two potential alternatively spliced exon-containing PCR products were identified in the three tissue sets assayed (Figure 1). Sequence analysis of these bands confirmed the presence of two alternatively spliced exons, both of which had previously been identified in human myometrium. The first was a 132 bp spliced exon located in the S0-S1 linker region identified by Korovkina et al. [26], and the second was an 87 bp spliced exon located in the S8-S9 linker region identified by Wallner et al. [10]. PCR of reverse transcriptase negative controls (RT-) and a water control (no cDNA template) did not generate any products, confirming the absence of genomic DNA contamination (data not shown).

### Quantitative analysis of alternatively spliced exon expression using real-time PCR

Quantitative analysis of alternatively spliced exon expression was performed using sequence-specific hydrolysis TaqMan probes to analyse mRNA expression of these exons in non-pregnant myometrium and pregnant myometrium, prior to and after labour onset, as outlined. In order to correct for random errors from sources such as pipetting inaccuracies, separate real-time PCR reactions were performed in triplicate for each amplicon involved. Agarose gel electrophoresis, as well as sequencing analysis, confirmed the specificity of PCR products formed, yielding single product bands of the expected size (data not shown).

**Figure 2:**
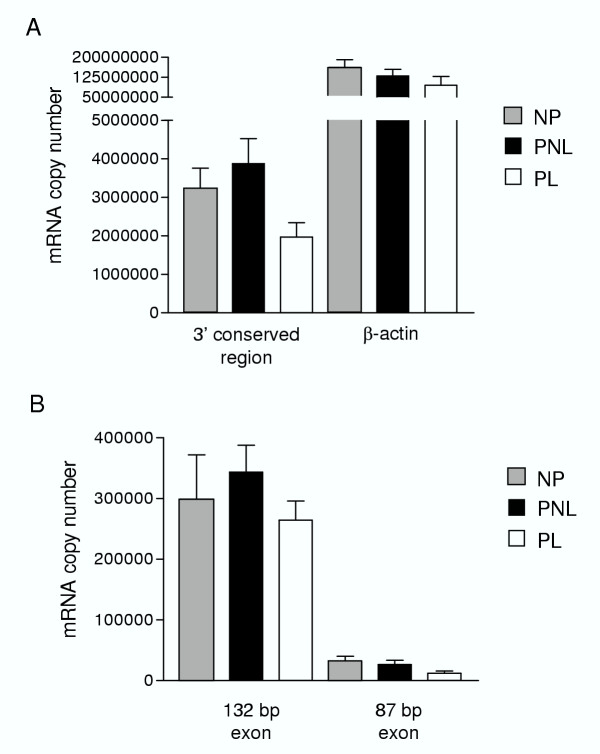
**Maxi-K α subunit mRNA expression analysed by quantitative real-time PCR**. Results shown represent mean (± standard error of the mean, SEM) copy number values for (**A**) total Maxi-K α subunit (represented by the 3' conserved region), and β-actin, and (**B**) the 132 bp and 87 bp alternatively spliced exons of the Maxi-K α subunit. Copy number values were obtained based on product-specific serially-diluted cDNA standards, generated individually for each amplicon of interest. Tissue sets are indicated by striped columns (non-pregnant, NP), black columns (pregnant not-in-labour, PNL) and open columns (pregnant in-labour, PL).

Quantitative results for each amplicon were obtained by determination of the threshold cycle (C_T_) values for each sample, as determined mathematically by the "Second Derivatives" method. Mean absolute cDNA copy number values for each probed amplicon involved, in each myometrial sample, were calculated and grouped per tissue set (i.e. NP, PNL, PL), as shown in Figure 2. All data were normally distributed, as determined by Normality plots for each group (P > 0.05). Analysis of the expression of the housekeeping gene, β-actin, showed no significant differences between the three tissue sets assayed (P > 0.05). The 3' conserved region of the Maxi-K α subunit was analysed as a measure of overall α subunit expression. The results of this analysis indicated a decrease in expression of the α subunit transcript with labour onset (Figure 2A). Although this did not reach statistical significance (P = 0.052), the observed decrease in α subunit mRNA expression at labour is in agreement with the decrease seen in α subunit protein levels at labour, reported recently [27]. Quantitative analysis of the expression of the 132 bp and 87 bp spliced exon transcripts indicated no significant differences in expression, in absolute terms, between NP, PNL, and PL tissues (P > 0.05)(Figure 2B).

**Figure 3:**
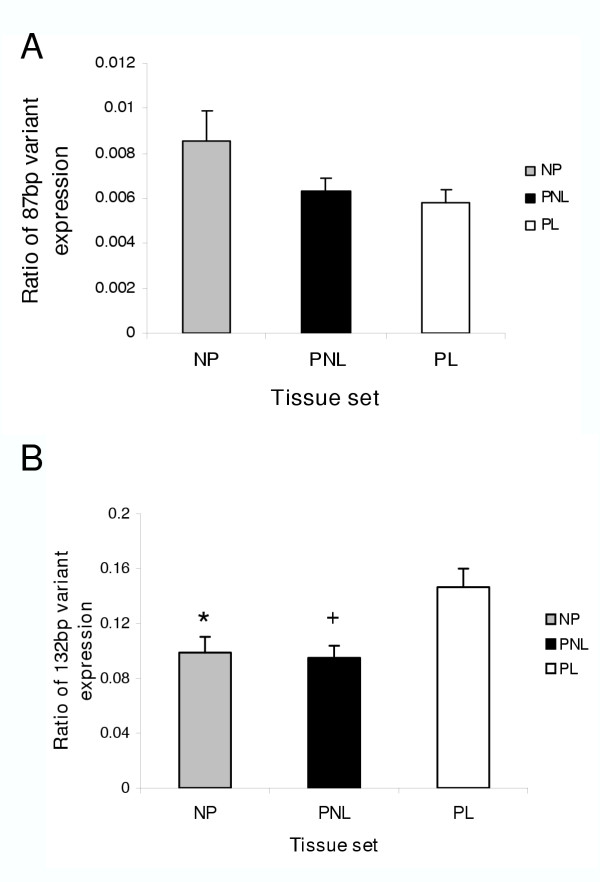
**Expression of 132 bp and 87 bp variant mRNAs of the Maxi-K α subunit**. The histograms show the mean (± standard error of the mean, SEM) of the ratios of spliced exon to total maxi-K α subunit mRNA for both (**A**) the 87 bp and (**B**) the 132 bp variants in NP, PNL and PL tissues. Results indicate significantly higher expression of the 132 bp exon as a proportion of the total α subunit with labour onset, compared to non-pregnant and pregnant not-in-labour samples. There is no change in expression of the 87 bp variant between the three tissue sets. Tissue sets are indicated by striped columns (non-pregnant, NP), black columns (pregnant not-in-labour, PNL) and open columns (pregnant in-labour, PL). *P < 0.05 versus PL; ^+^P < 0.01 versus PL.

The results of expression analyses for the 87 bp and 132 bp spliced exons as a proportion of the total Maxi-K α subunit (i.e. the 87 bp and 132 bp variants) are demonstrated in Figure 3. Analysis of mRNA expression of the 87 bp variant indicated no significant differences between the three tissue sets assayed (Figure 3A). Expression of this variant mRNA accounted for only 1% of total Maxi-K α subunit expressed. However, the proportion of Maxi-K channels expressing the 132 bp spliced exon was significantly increased with labour onset (PL), compared to both non-pregnant (NP)(P < 0.05) and pregnant not-in-labour myometrial tissues (PNL) (P < 0.01)(Figure 3B). Duplicate RT and quantitative PCR analysis confirmed these data. The increase in proportion of this 132 bp variant could be equated to approximately 1.7 fold, from 9% to 15% of total α subunit mRNA expressed.

## Discussion

In this study RT-PCR analyses were performed to identify mRNA expression of the Maxi-K α subunit, and alternatively spliced exons of this subunit, in human myometrium in its non-pregnant state, and at term pregnancy, prior to and after labour onset. This was followed by quantitative real-time PCR, which was performed to determine the overall pattern of expression of the Maxi-K α subunit as well as expression of alternatively spliced exons of this subunit identified in these tissue sets.

Our findings indicate a trend towards a decrease in α subunit mRNA levels with human labour onset. In order to maintain the uterus in a quiescent state during pregnancy, K^+ ^channels provide a potent repolarizing current through the efflux of K^+ ^ions, thereby dampening cell excitability and promoting cell relaxation [32]. Previous studies on murine and rodent myometrium have reported conflicting results for Maxi-K α subunit mRNA and protein expression during pregnancy and with labour onset. Song et al. [26] identified a decrease in Maxi-K α subunit protein levels in rats at term pregnancy, whereas Benkusky et al. [19] indicated an increase in protein levels of this subunit in mouse term myometrium. However, a recent report has outlined significant down-regulation in the protein levels of both α- and β-subunits of the Maxi-K channel in human myometrium at labour onset, suggesting that the loss of Ca^2+ ^and voltage sensitivity is at least partly due to decreased levels of the Maxi-K channel [27]. Our findings are in agreement with this report, with the highest levels of mRNA expression in the PNL group, and decreased mRNA expression of the Maxi-K α subunit with labour onset (Figure 2). Although the decrease in mRNA expression between PNL and PL tissues was ~50%, it was found not to be statistically significant (P = 0.052). A reduction in expression of the Maxi-K α subunit could allow for enhanced myometrial contractility, as reduced α subunit expression would permit an increase in intracellular Ca^2+ ^levels without the activation of an opposing K^+ ^conductance [32].

Maxi-K channels derive their molecular diversity by alternative splicing of their α subunit transcript at several key sites, which generate channel variants with distinct phenotypes [7,15,16]. Previous studies provide evidence that alternate splicing effects calcium and voltage sensitivity of the maxi-K channel and thus channel function in myometrium [16], surface expression [17], and sensitivity to protein phosphorylation of the maxi-K channel [18]. Further direct evidence for the role of alternate splicing of the maxi-K transcript in altering maxi-K protein function in myometrium is provided by the finding of up-regulation of maxi-K splice variants known to alter channel current through alterations in calcium and voltage sensitivity in pregnant mouse myometrium [19]. Our results from RT-PCR analysis indicate the presence of only two spliced exons, both of which had been identified previously, in human myometrium. The 132 bp exon, previously identified by Korovkina et al. [28], encodes a 44 amino acid peptide that is inserted into the first intracellular loop of the Maxi-K α subunit, and contains four potential consensus sites for post-translational modification. The 87 bp exon, isolated by Wallner et al. [10], encodes a 29 amino acid peptide that is introduced into the loop region between hydrophobic regions S8 and S9 of the α subunit protein. Protein sequence analysis of this exon using PROSITE revealed a potential cAMP-/cGMP-protein kinase phosphorylation site (KKeT).

Quantitative real-time PCR was used to determine whether identified spliced exons displayed altered expression in pregnancy and/or with labour onset. The use of hydrolysis TaqMan probes provided high reaction specificity and sensitivity, and allowed for highly accurate quantification of target sequences. Our results indicate that there was no significant change in expression of the 87 bp spliced exon, in absolute terms, in non-pregnant (NP), pregnant not-in-labour (PNL) and pregnant in-labour (PL) myometrium. Furthermore, analysis of the expression of this spliced exon as a proportion of the total α subunit expressed (i.e. the 87 bp exon-containing α subunit splice variant) also showed no differences between the three tissue sets assayed. The proportion of Maxi-K α subunits expressing this variant was very low, accounting for only ~1% of Maxi-K channels expressed in the three tissues sets. Little is currently known about the physiological effects of expression of this variant of the Maxi-K channel. However, as described above, it contains a consensus sequence for protein kinase phosphorylation; therefore, it may have important consequences for post-translational modification of channel function. Also, the region into which this exon is inserted, between hydrophobic regions S8 and S9 of the α subunit protein, is thought to be involved in determining the Ca^2+ ^sensitivity of the Maxi-K channel [16].

In contrast, while there was no significant change in the mRNA levels of the 132 bp spliced exon in absolute terms between the three tissue sets, there was a significant increase (~1.7 fold) in the proportion of Maxi-K α subunits expressing this exon with labour onset, compared to both non-pregnant and pregnant not-in-labour tissues. Messenger RNA for this 132 bp variant was expressed at much higher levels in comparison to the 87 bp variant, accounting for 9% of total Maxi-K channels in NP and PNL tissues, increasing to 15% at labour onset. An increased proportion of myometrial Maxi-K α subunits expressing the 132 bp spliced exon with labour onset in the human is of interest, as the presence of this exon has been shown to decrease both the Ca^2+ ^and voltage sensitivities of the Maxi-K channel [28]. Thus, our findings may provide an additional explanation for the observation of decreased Ca^2+ ^and voltage sensitivities of Maxi-K channels after labour onset [24] to reduced expression levels of the α- and β-subunits reported recently [27]. The exact mechanism by which the presence of this exon causes decreased sensitivity of the Maxi-K channel is unknown. It is possible that post-translational modifications at the four consensus sites present in the exon, perhaps in combination with conformational changes in the intracellular loop due to its increased length, may bring about the observed changes. Determination of the precise mechanism by which the 132 bp exon causes decreased channel sensitivity is an interesting question and is the subject of ongoing investigations in our laboratory.

## Conclusions

Following the onset of labour, the putative disabling of the link between Ca^2+ ^and Maxi-K channel activation would permit Ca^2+ ^levels in the cell to rise without the activation of an opposing K^+ ^conductance, hence increasing the availability of Ca^2+ ^for myometrial contraction [30]. Our findings here suggest that, in human myometrium at labour onset, in addition to decreased Maxi-K α subunit mRNA expression, the increased proportion of Maxi-K α subunits containing the 132 bp spliced exon that are insensitive to Ca^2+ ^and voltage levels, is responsible for enhanced uterine activity at the time of labour. Whether these variants are assembled as homo- or hetero-tetramers at the plasma membrane remains to be determined. Further investigations are required to assess factors such as the role of β-subunit attachment and modulation of the calcium bowl, and their input to regulation of Maxi-K channels during human pregnancy and labour. This study provides the first quantitative analysis of Maxi-K α subunit mRNA expression in human myometrium, and also highlights alternative exon splicing as a potentially important control mechanism by which myometrial Maxi-K channels may be modulated to suit their functional requirements during these physiological processes.

## Authors' Contributions

MC designed the quantitative RT-PCR techniques and carried out all experimental work. JJM recruited patients, organised the collection of tissues, and conceived of the study. TJS conceived of the study, and participated in its design and coordination. All authors read and approved the final manuscript.
